# Home-Based Transcutaneous Neuromodulation Improved Constipation via Modulating Gastrointestinal Hormones and Bile Acids

**DOI:** 10.1155/2018/2086163

**Published:** 2018-04-29

**Authors:** Zhenyang Ge, Zhijun Duan, Hang Yang, Shengai Zhang, Shuang Zhang, Lixia Wang, Dong Yang, Xiaoyu Sun, Zhifeng Zhang, Liping Su, Hong Zhu, Dongdong Zhou, Bojia Liu, Honggang Shi, Jun Yu, Hui Yang, Qingyong Chang, Nina Zhang, Dongsheng Wu, Jiande D. Z. Chen

**Affiliations:** ^1^Neurogastroenterology and Motility Center of China-US Cooperation, The Second Department of Gastroenterology, College of Integrative Medicine, The First Affiliated Hospital of Dalian Medical University, No. 222 Zhongshan Road, Dalian, Liaoning 116011, China; ^2^Clinical Laboratory, The First Affiliated Hospital of Dalian Medical University, No. 222 Zhongshan Road, Dalian, Liaoning 116011, China; ^3^The Second Department of Neurosurgery, Affiliated Zhongshan Hospital of Dalian University, No. 6 Jiefang Street, Dalian, Liaoning 116001, China; ^4^Department of Gastroenterology, Nanjing Drum Tower Hospital, No. 321 Zhongshan Road, Nanjing, Jiangsu 210008, China; ^5^The School of Engineering and Materials Science, Queen Mary, University of London, London E1 4NS, UK; ^6^Division of Gastroenterology and Hepatology, Johns Hopkins Center for Neurogastroenterology, Baltimore, MD 21224, USA

## Abstract

This study aims to investigate the role of transcutaneous neuromodulation (TN) on the regulation of gastrointestinal hormones and bile acids in patients with functional constipation (FC). Twenty FC patients were treated with TN for four weeks. The effects of TN on symptoms were evaluated by questionnaires. Plasma levels of serotonin (5-HT), motilin, somatostatin, and vasoactive intestinal peptide (VIP) were measured by ELISA and 12 individual bile acids assayed by liquid chromatography tandem mass spectrometry. Results were as follows. (1) TN treatment increased the frequency of spontaneous bowel movement, improved the Bristol Stool Score, and reduced Patient Assessment of Constipation Symptom score and Patient Assessment of Constipation Quality of Life score. (2) FC patients showed decreased plasma levels of 5-HT, motilin, and VIP and an increased plasma level of somatostatin (*P* < 0.05). Four-week TN treatment increased plasma levels of 5-HT and motilin and decreased the plasma level of somatostatin in the FC patients (*P* < 0.05). (3) Taurocholic deoxycholate, taurocholic acid, and taurocholic lithocholic acid were increased in the FC patients (*P* < 0.005) but reduced by TN treatment (*P* < 0.05). This study has suggested that the therapy may improve the symptoms of FC by alleviating the disorders of gastrointestinal hormones and bile acids.

## 1. Introduction

Functional constipation (FC) is mainly characterized by a low defecation frequency, defecation difficulty, and incomplete defecation. The prevalence of FC has been high in female and elderly [1]. Based on the pathophysiology, FC is classified as follows: Slow Transit Constipation (STC), Defecatory Disorder (DD), and Normal Transit Constipation (NTC) [2]. Traditional treatment is mainly focused on drugs such as leavening agent, osmotic laxatives, stimulant laxatives, prosecretory agents, and prokinetic agents [3]. Recently, the bile acid regulator has become a widely applied medical therapy [4]. However, there are still a large number of patients who are refractory to medical therapies and there is a need to develop effective therapies for FC.

Neuromodulation has recently been introduced for the treatment of FC, such as sacral nerve stimulation [5] and tibial nerve stimulation [6] with inconclusive results and largely unknown mechanisms. In a previous study, we reported a promising ameliorating effect of transcutaneous neuromodulation (TN) in FC patients [7]. In this method, electrical stimulation was delivered noninvasively via surface electrodes placed at both an acupoint ST36 and the posterior tibial nerve using an external watch-size stimulator. The therapy was home-based and self-administrated. The therapeutic effect of TN was reported to be mediated via the autonomic functions (enhancement of vagal activity and suppression of sympathetic activity) and hypothesized to improve gastrointestinal motility. However, its exact prokinetic mechanisms, especially the involvement of neurotransmitters and bile acids, were still unclear.

Acupuncture and electroacupuncture have been reported to alter various neurotransmitters in both patients with functional gastrointestinal diseases and animal models of constipation, such as serotonin (5-HT) [8, 9], motilin [10], and vasoactive intestinal peptide (VIP) [11, 12]. While little is known about the direct effects of acupuncture or electroacupuncture on bile acid metabolism, which is involved in 5-HT activation [13]. Meanwhile, the abnormal metabolism of bile acids was found in patients with FC [14, 15], indicating their potential roles in colon motility and hormone secretion to influence intestinal transit [16].

Accordingly, this experiment aims to investigate the therapeutic effect of the home-based, noninvasive, and self-administrated TN at ST36 and posterior tibial nerve on FC and to explore its mechanisms involving neurotransmitters associated with colon motility and bile acids in patients with FC.

## 2. Materials and Methods

### 2.1. Study Subjects

This study included 20 FC patients and 20 healthy volunteers, recruited from the Department of Physical Examination, the First Affiliated Hospital of Dalian Medical University from December 2016 to April 2017. The study protocol was approved by the hospital ethics committee (number LCKY2016-31) and registered in Chinese Clinical Trial Registry (number ChiCTR-OOC-16010259). All participants in the study signed the informed consent form and were free to quit the study at any time for any reasons.

#### 2.1.1. Inclusion Criteria for Patients

Inclusion criteria for patients were as follows: (1) aged 18–80 years; (2) met Rome IV criteria [2] for FC; (3) no organic diseases by colonoscopy; (4) no acupuncture treatment in the preceding 3 months; (5) no participation in any clinical trials in the preceding 3 months; and (6) being capable of conducting the treatment at home.

#### 2.1.2. Exclusion Criteria

Exclusion criteria were as follows: (1) abdominal surgery history; (2) metabolic diseases such as diabetes and hypothyroidism; (3) neurologic diseases or any organic diseases causing constipation such as multiple sclerosis, rachischisis, Parkinson's disease, or spinal cord injury; (4) any organic diseases, such as liver, gallbladder, pancreas, or intestines; (5) pregnancy or intention to become pregnant during the trial; (6) allergic to skin preparation or electrodes; (7) being with an implanted pacemaker; (8) Self-Rating Anxiety Scale (SAS) score > 60 or/and Self-rating Depression Scale (SDS) score > 63; and (9) refusal of blood withdrawing.

#### 2.1.3. Inclusion Criteria for Healthy Volunteers

Inclusion criteria for healthy volunteers were as follows: (1) aged 18–80 years; (2) no diagnosis of gastrointestinal diseases in the preceding 3 months and no constipation and other gastrointestinal symptoms; (3) no abdominal surgery, metabolic disease, neurologic diseases, or other organ diseases; (4) no medications affecting bowel movement in the preceding 3 months; (5) willingness to sign the informed consent; and (6) willingness for blood withdrawing.

### 2.2. Experimental Methods

#### 2.2.1. TN Treatment

**Figure 1:**
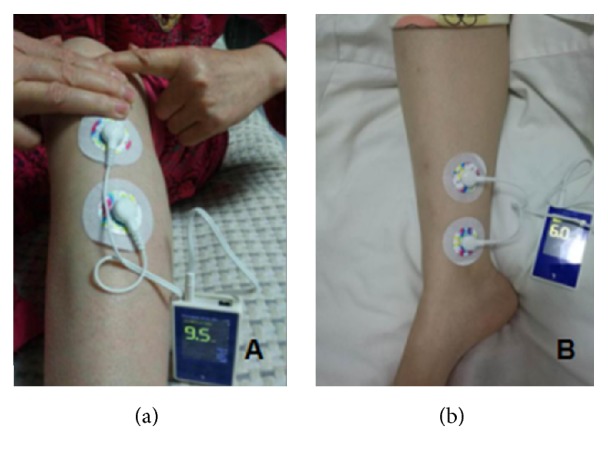
*The location of electrode*. (a) ST36: one electrode was placed at ST36 and the other at 4 cm below ST36 along the same meridian. (b) Posterior tibial nerve: one electrode placed at approximately two fingers' breadth up to the malleolus and posterior to the tibia and the other at 4 cm above the first electrode.

A watch-size microstimulator, called Neuromodulator for Gastrointestinal Functions (SNM-FDC01, Ningbo Maida Medical Device Inc., Ningbo, China), was used for the TN treatment via electrodes placed at the posterior tibial nerve and the acupoint ST36 (Zusanli, either right leg or left leg) according to a previous study [7]. For the ST36, one electrode was placed at ST36 and the other at 4 cm below ST36 along the same meridian; for the posterior tibial nerve, one electrode was placed at approximately two fingers' breadth up to the malleolus medialis and posterior to the tibia and the other at 4 cm above the first electrode (Figure 1). The following parameters were used for the TN: train on-time of 2 sec and off-time of 3 sec, pulse width of 0.5 ms, pulse frequency of 25 Hz, and amplitude of 2–10 mA (at the maximum level tolerated by the subject) [7].

#### 2.2.2. Experimental Protocols

**Figure 2:**
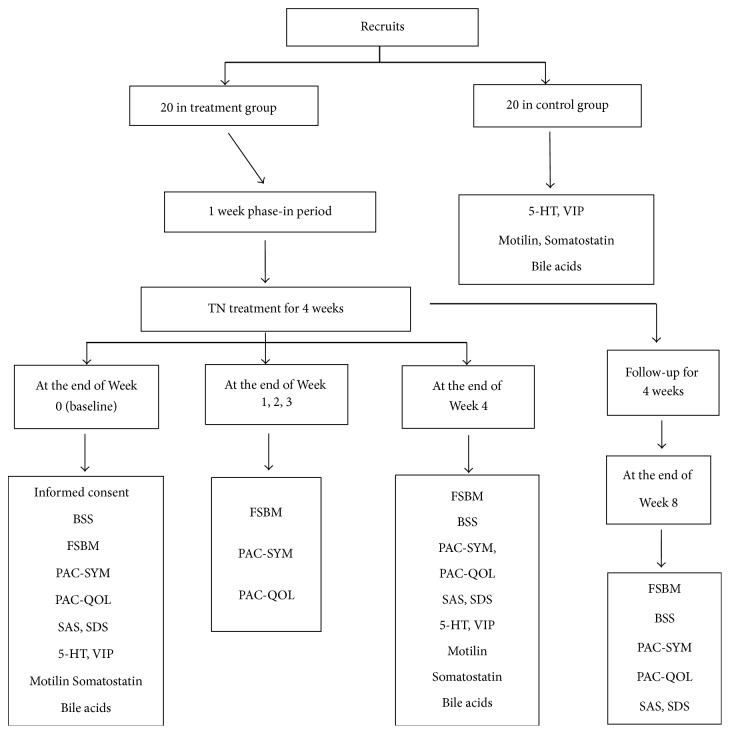
Experimental protocols.

The TN treatment was performed at home and administrated by the patient. Before the initiation of the treatment, each patient was trained for the use of the therapy, including identification of stimulation locations, preparation of the skin, operation of the device, and recharging of the device as well as the completion of questionnaires. Before the TN treatment, there was a phase-in period of one week during which the patient was required to stop taking any medications affecting gastrointestinal motility or defecation. The TN treatment was given twice a day: 6–8 am before breakfast and 6–8 pm after dinner, each lasting 1 hour. Various questionnaires were completed by the patient at the end of each week, including the phase-in period (Figure 2). At the end of the 4-week treatment, the patients were informed to come to the hospital to return the stimulation device and at the meantime blood samples were taken for the assessment of gastrointestinal hormones and bile acids. Four more weeks (no TN treatment) later, the patients were informed to complete the symptom questionnaire for the assessment of possible sustained effects of TN treatment; then all the questionnaires were collected.

During the treatment, participants should avoid using other cathartic in principle, unless no defecation lasted 3 days or more. Under this condition, Enema Glycerini was allowed to assist defecation once or twice and had to be recorded in the defecation diary. Emergency medicine could also be applied during the phase-in period. Participants were free to withdraw from the experiment for further treatment if they kept suffering from constipation.

No sham treatment was given in this study because firstly the ameliorating effect of the TN on constipation was previously established in a previous placebo-controlled study [7] and the major aim was to investigate the underlying mechanisms of TN treatment; secondly, it is well-known that the placebo effect is rare in objective physiological or mechanistic measurements.

#### 2.2.3. Assessment of Constipation-Related Symptoms

Constipation-related symptoms were assessed by patient completed questionnaires which included (a) Bristol Stool Score (BSS) [17], (b) the frequency of spontaneous bowel movement per week (FSBM), (c) Patient Assessment of Constipation Symptom (PAC-SYM) [6]; (d) Patient Assessment of Constipation Quality of Life (PAC-QOL) [6], (e) SAS [18], and (f) SDS [18]. Before the treatment (baseline), items (a)–(f) were completed. During the first 3 weeks of treatment, items (b)–(d) updated the defecation diary and the time and frequency of the use of medications were recorded. At the end of the 4-week treatment, items (a)–(f) were completed. Four more weeks (no TN treatment) later, items (a)–(f) were completed.

#### 2.2.4. Quantification of Gastrointestinal Hormones and Bile Acids

Blood samples were taken from FC patients and healthy volunteers for the measurement of levels of plasma gastrointestinal hormones and bile acids. Healthy volunteers were not treated with TN. A 4 ml blood sample was taken in fasting state (7-8 am) from the patients before and at the end of the 4-week treatment. A 4 ml blood sample was also taken from each healthy volunteer in fasting state. The sample was collected in a test tube containing EDTA, placed for 30 minutes under room temperature and centrifuged for 10 minutes at 4000 r/min; then 1.5 ml upper plasma were transferred into an EP tube and saved in a −80°C refrigerator.

The enzyme-linked immunosorbent assay method was applied to measure the plasma levels of gastrointestinal hormones, including 5-HT, motilin, somatostatin, and VIP according to the manufacturers instructions (USCN Life Science Inc., Wuhan, China).

Liquid chromatography tandem mass spectrometry [19] was used to assay 12 subtypes of bile acids (Dalian Meilun Biotech Co., Ltd., Dalian, China), including tauroursodeoxycholic acid (TUDCA), taurocholic acid (TCA), taurochenodeoxycholic acid (TCDCA), taurocholic deoxycholate (TDCA), chenodeoxycholic acid (CDCA), ursodeoxycholic acid (UDCA), cholic acid (CA), lithocholic acid (LCA), taurocholic lithocholic acid (TLCA), glycochenodeoxycholic acid (GCDCA), glycocholic acid (GCA), and glycodeoxycholic acid (GDCA).

### 2.3. Statistical Analysis

Statistical analysis was conducted using SPSS16.0 Software. Data with a normal distribution are presented as mean ± SE. Data that did not have a normal distribution are presented as median (quartile range). The Independent Samples *t*-test was used to compare general characteristics of groups. Wilcoxon Signed Ranks Test was used to compare the measurements before and after the treatment, while Mann-Whitney* U* Test was used to compare the measurements between treatment group and control group. The correlation analysis was performed using the Spearman correlation method. Statistical significance was assigned for *P* < 0.05.

## 3. Results

A total of 20 FC patients (14 female, 6 male) were enrolled in the study. Three patients were dropped out during the study: two of them attributed to allergic reaction to the stimulation electrodes and the other due to poor compliance (incomplete questionnaires). The remaining 17 patients underwent the trial without any uncomfortable complaints. The ratio of female/male, age, and body mass index of the patients were 14/6, (49.30 ± 11.94), and (23.17 ± 3.64), respectively. Those of the healthy controls were 12/8, (47.70 ± 14.71), and (23.31 ± 2.44), respectively. No ratio and mass index difference was noted between the patients and controls (*P* > 0.05).

### 3.1. Improvement in Constipation by TN

The 4-week TN treatment significantly improved the frequency of spontaneous bowel movement per week. The FSBM was significantly increased in comparison with the baseline at the end of the 3rd and 4th weeks of the treatment (*P* < 0.001). Most interestingly, the FSBM increase remained significant at 4-week follow-up without stimulation (*P* < 0.001), suggesting a sustained effect after TN which was not previously reported [7].

The stool characteristics assessed by the Bristol Stool Score was also significantly improved with the treatment. Types 1 and 2 in the BSS are hard and suggestive of constipation, types 3–5 are considered normal, and types 6 and 7 represent loose and liquid stools associated with diarrhea [17]. After the 4-week TN treatment, the score of BSS was increased from a median of 2.0 (1.0~2.0) at the baseline level to a median of 3.0 (3.0~4.0) (*P* < 0.001; see Table 1) and remained at a median of 3.0 (3.0~3.0) after 4 weeks of follow up without TN treatment (*P* < 0.001 versus baseline).

The TN treatment also significantly improved constipation symptoms and the quality of life. Compared with the baseline, the PAC-SYM and the PAC-QOL were significantly decreased at the end of the 3rd and 4th week of the treatment (*P* < 0.001) and remained reduced after 4 weeks of follow-up without the treatment (*P* < 0.001 versus baseline; see Table 1). The PAC-SYM score was decreased from a median of 26.0 (24.5~32.0) at the baseline level to a median of 16.0 (14.0~18.5) (*P* < 0.001) after the 4-week treatment and a median of 18.0 (14.5~18.5) at the end of 4-week follow-up without the treatment (*P* < 0.001 versus baseline). The PAC-QOL score was decreased from a median of 52.0 (48.0~58.0) at the baseline level to a median of 31.0 (27.0~36.0) (*P* < 0.001) after the 4 weeks' treatment and a median of 33.0 (26.5~38.0) at the end of 4 weeks' follow-up after stopping the treatment (*P* < 0.001 versus baseline).

### 3.2. Alteration in Plasma Gastrointestinal Hormones

**Figure 3:**
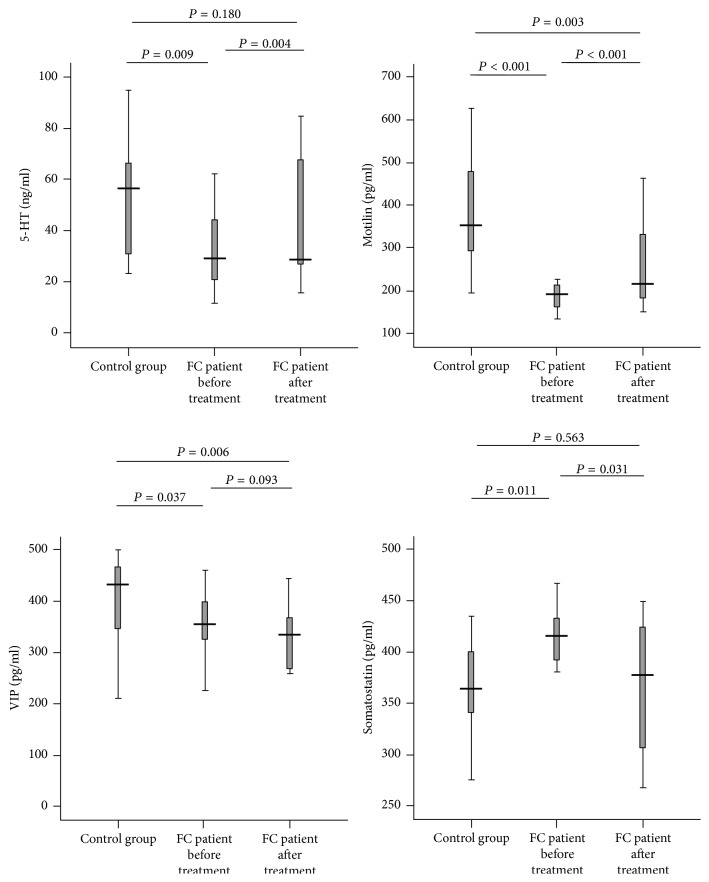
*The levels of the plasma gastrointestinal hormones in FC patients and control group*. FC patients showed a decreased plasma level of 5-HT, motilin, and VIP and an increased plasma level of somatostatin (all; *P* < 0.05 versus control group). The 4-week TN treatment increased plasma levels of 5-HT and motilin and decreased the plasma level of somatostatin in FC patients (*P* < 0.05 versus before treatment).

A number of major gastrointestinal hormones associated with motilin were altered in FC patients and improved after 4-week TN treatment. As shown in Figure 3, (1) the plasma level of 5-HT in the FC patients before TN therapy was lower than that of the healthy volunteer control group (*P* = 0.009) but increased significantly after the treatment (*P* = 0.004 versus baseline; *P* = 0.180 versus controls), (2) the concentration of the plasma motilin in FC patients was lower than that of the control group (*P* < 0.001) but increased significantly after the treatment (*P* < 0.001) although being still lower than that of the control group (*P* = 0.003), (3) the plasma level of VIP in FC patients was lower than that of the control group (*P* = 0.037) and not altered by TN treatment (*P* = 0.093 versus baseline; *P* = 0.006 versus controls), and (4) the plasma level of somatostatin in the FC patients was higher than that of the control group before the TN therapy (*P* = 0.011) but increased after the 4-week therapy (*P* = 0.031 versus baseline; *P* = 0.563 versus controls).

### 3.3. Modulation of Bile Acid Metabolism

**Figure 4:**
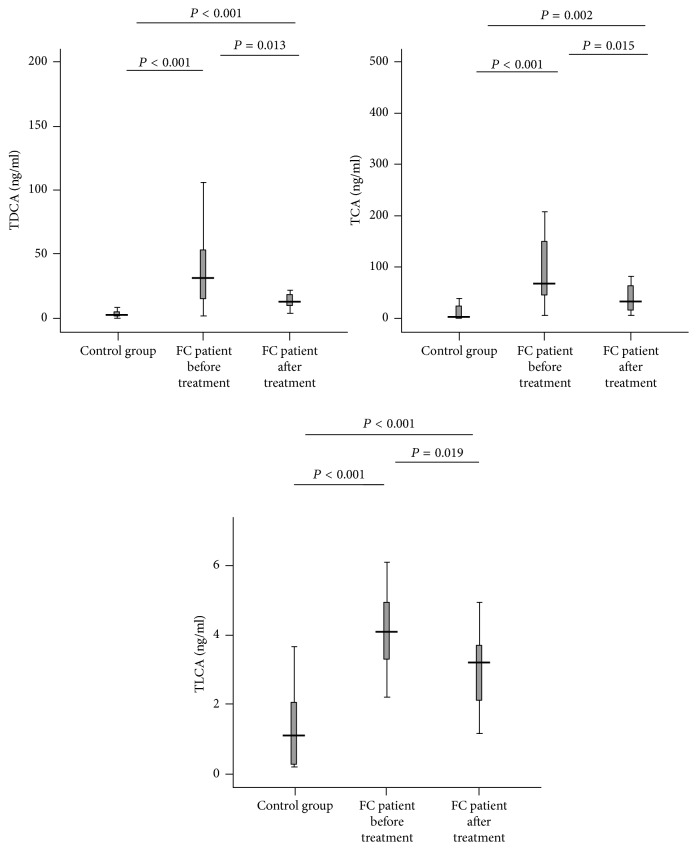
*The levels of the plasma bile acids in FC patients and control group*. TDCA, TCA, and TLCA were increased in FC patients (all; *P* < 0.005 versus control group) but reduced by TN treatment (all; *P* < 0.05 versus before treatment).

Plasma levels of three of 12 subtypes of bile acids, TDCA, TCA, and TLCA were significantly higher in FC patients before treatment than that of healthy volunteers (control group) (all, *P* < 0.005 versus controls) but decreased obviously after 4-week TN treatment, with TDCA and TCA close to the control levels (all; *P* < 0.05 versus baseline) (Figure 4). For other bile acids, TUDCA, TCDCA, UDCA, CDCA, GCA, GCDCA, GDCA, and CA, we did not see any difference between FC patients and healthy controls and any change after TN treatment (Table 2).

### 3.4. Correlation Analysis of the Plasma Gastrointestinal Hormones and Bile Acids (Table 3)

There was no correlation between the plasma gastrointestinal hormones (5-HT, motilin, VIP, and somatostatin) and bile acids (TDCA, TCA, and TLCA) in the control group and treatment group, neither before nor after TN treatment (*P* > 0.05).

### 3.5. Correlation Analysis of the Plasma Gastrointestinal Hormones, Bile Acids, and the Scores of Questionnaires (Table 4)

There was no correlation between the plasma gastrointestinal hormones (5-HT, motilin, VIP, and somatostatin), bile acids (TDCA, TCA, and TLCA), and the scores of questionnaires (FSBM, BSS, PAC-SYM, and PAC-QOL) in FC patients before TN therapy (*P* > 0.05).

## 4. Discussion

Demonstrated by questionnaires of PAC-SYM and PAC-QOL TN treatment significant improved constipation symptoms and life qualities, which is consistent with the previous study [7]. More interestingly, we found that TN increased FC patients' plasma levels of 5-HT and motilin and decreased the plasma level of somatostatin. Furthermore, plasma levels of TDCA, TCA, and TLCA were downregulated by TN. Nevertheless, we did not find significant correlations between the gastrointestinal hormones and subtype bile acids in FC patients or between the above indexes and the scores of questionnaires.

It has been reported that patients with FC have a trend to suffer from mental or psychogenic diseases, such as anxiety-depression status [20–22]. Thus, in this study we introduced questionnaires of SAS and SDS and found that the FC patients were indeed in various degrees of anxiety-depression status. Therefore “SAS < 60, SDS < 63” was chosen as inclusion criteria to exclude the FC patients with serious mental or psychogenic disorders. The results showed that TN therapy did not aggravate the anxiety-depression.

TN has gained much attention due to being noninvasive, home-based, and self-administred. Our previous study has included sham treatment as control, and the effectiveness of TN treatment on FC was confirmed. Our major aim in this study was to investigate the underlying mechanisms of TN in terms of gastrointestinal hormones and bile acids. So, we do not establish sham treatment.

It is clear that the plasma levels of gastrointestinal hormones secreted by enteroendocrine cells were changed by the TN. Enteroendocrine cells are controlled by enteric neurons, which govern all gastrointestinal functions, including motility. The enteric nervous system receives information from the parasympathetic and orthosympathetic nervous systems [23–25]. Our previous study reported that TN might improve the symptoms of FC patients by increasing vagal activity and concurrently decreasing sympathetic activity [7]. The animal experiment also demonstrated that both electroacupuncture and transcutaneous electroacupuncture at ST-36 were able to ameliorate intestinal hypomotility by increasing vagal activity [26]. Therefore, we believed that gastrointestinal hormones played an important role in the regulation of gastrointestinal motility.

5-HT is important to regulate gut motility and locates in the subsets of mucosal cells and neurons of gut [27]. 5-HT has many receptors expressed widely in gut, and the receptors of 5-HT3 and 5-HT4 their clinical value in regulating the gut motility has been proved [28]. Prucalopride is a 5-HT4 agonist which was found effective and safe for constipation patients [29, 30]. Researchers found that patients with STC may have 5-HT transporter gene which interferes with 5-HT reuptake [31]. In this study we found that the concentration of 5-HT in FC patients was lower and increased significantly after 4 weeks of TN treatment. Xu et al. [8] used electroacupuncture to stimulate Tianshu, Fujie, and Shangjuxu to treat serious FC. They found that plasma 5-HT increased and NOS decreased significantly after therapy, which is consistent with our results, and the similar effects were also shown when the therapy was applied on animals [9].

Motilin is secreted by Mo cells mainly distributed in digestive system [32], which is considered to contribute to gastrointestinal tract contraction [33]. We found that motilin's concentration in FC patients is lower than normal but increased after the TN therapy. The early study indicated that motilin level in STC group was higher than normal [34]. However, more researches suggested motilin level in FC patients is lower than that in healthy individuals, which is consistent with our study [35, 36]. Interestingly, Fei et al. [37] detected the motilin level in descending colon and rectal mucosa and also found it significantly decreased in STC group.

VIP is a kind of neural polypeptide scattering in the nervous system, immune system, and digestive system, which is composed of 28 acid residues and secreted by neurons, endocrine, and immune cells. It can regulate gastrointestinal physiological functions [38]. King et al. [39] found that 1/3 of STC children had lower VIP nerve fiber density in the proximal colon. Tomita [40] found that STC patients' colon had a weaker response to VIP than normal colon. Xiong et al. [41] found that different frequency of electroacupuncture at Quchi point and ST36 could elevate the VIP level in serum of FC patients and relieve the symptoms. This study showed that the plasma level of VIP in FC patients was lower than that of control group, and 4-week therapy did not cause significant change. In this study, the cases could not be subtyped based on constipation characteristics due to small sample size. In addition, animal experiments indicated that the distribution of VIP positive nerve fibers had no difference between STC and normal mice [42].

Somatostatin is a kind of cyclic peptides distributing in nervous system and digestive system. Previous researches mainly focused on its influences on central nervous system. Recent reports implicate a negative effect on gastrointestinal peristalsis and hormones secretion [43]. In this study, we found that somatostatin in FC patients was higher than that in control group and decreased after TN therapy. Xi et al. [44] found somatostatin level in plasma increased as colon movement weakened in 90 healthy individuals. Further study showed serum somatostatin in STC patients raised obviously compared to normal controls [45]. Zhao et al. [46] found that* Lactobacillus* could relieve constipation in mice and decrease the somatostatin level, which was in line with findings of other researches [24, 47]. It was also reported that acupuncture and electroacupuncture improved symptoms of patients with constipation, with significantly decreased serum somatostatin [48].

It is well-known that bile acid in small intestine is mainly responsible for the fat emulsion, lipid digestion, and absorption, while some other physiological functions such as antimicrobial activity, gastrointestinal movement regulation, water and electrolyte absorption, intestinal epithelium growth, and epithelial gene expression are gradually unveiled [49]. It has been demonstrated that the abnormal metabolism of bile acid is involved in the pathophysiological mechanism of chronic constipation [50, 51]. Bile acids can connect the G-protein coupled plasma membrane receptor on colonic epithelial cell and induce the secretion of electrolyte and water, which can also reinforce the colonic movement and stimulation of defecation [52]. Oddsson et al. [53] found that the bile acids induced secretion of more rectal fluids in irritable bowel syndrome (IBS) patients than healthy volunteers. The phasic contraction of colon was stimulated by infusion of 5 mmol/L or higher concentration of bile acids, but such high concentration cannot exist in colon physiologically unless the ileum is removed [54, 55]. It has been suggested that CDCA in rectum could effectively stimulate the proximal colon to produce pressure wave [56]. Furthermore bile acids have been shown to stimulate intestinal chromaffin cells, releasing 5-HT [13] which affects gastrointestinal dynamics. Bile acid regulators such as elobixibat have been emerging as novel therapeutics for chronic constipation. Thus, we further examined the subtypes of bile acids in the plasma and explored their relationship with gastrointestinal hormones.

We detected 12 subtypes of bile acids in the plasma and found that only the levels of TDCA, TCA, and TLCA in FC patients were higher than that of control group and decreased after TN therapy. We speculate that TN might regulate the bile acid metabolism in patients with constipation. Hofmann et al. [14] found that fecal bile acids of most FC children had a normal metabolism. However, some of them had abnormal bile acids metabolism. Their major fecal bile acid is 3-sulfate of CDCA, which can abolish CDCA secretory activity and may contribute to constipation. which implicated that abnormal metabolism of bile acids might be a cause of childhood FC. Shin et al. [15] have shown that IBS with diarrhea (IBS-D) patients had a higher fecal level of CA/CDCA in comparison with that of healthy control, whereas in IBS with constipation (IBS-C) patients lower fecal CDCA and DCA were found in feces than that in healthy control. The percentage of LCA in fecal excretion increased significantly in IBS-C group, which might be associated with slow transit. Abrahamsson et al. [57] found that the changes in bile acid synthesis might be associated with transmission of colon. Fast transit of colon could reduce the absorption of bile acid, while slow transit of colon could increase the absorption of bile acid. The different absorption led to more bile acid synthesis in IBS-D and less in IBS-C, suggesting a significant positive correlation between fecal bile acid and fecal weight [58]. However, in this trial, LCA in the plasma of FC patients was not detected which might be due to the excessive excretion and subsequently low absorption. In our study, the level of CDCA and CA were lower in FC patients but there was no statistical difference before and after the TN therapy. We have detected that TDCA, TCA, and TLCA were significantly higher in the FC patients before treatment than that of healthy volunteers control group and decreased obviously after TN treatment. These results indicate that TDCA, TCA, and TLCA were to be reabsorbed more in the bowel when patients suffered from constipation; then the absorption decreased as the constipation symptoms improved by TN therapy. Furthermore, the plasma bile acid spectrum may reflect the levels of the fecal bile acids partly, which deserve to be explored further. In addition, there was no obvious correlation between the plasma gastrointestinal hormones and bile acids. The exact link between bile acids and gastrointestinal hormones remains to be further investigated.

In conclusion, TN may improve the symptoms of FC patients by alleviating the disorders of gastrointestinal hormones and bile acids metabolism. Strategies aiming at modulating such disorders may emerge as novel therapeutics for chronic constipation.

## Figures and Tables

**Table 1:** Comparisons of FC patients' questionnaires. Median (quartile range), Wilcoxon Signed Ranks Test.

	0th week (baseline)	1st week	2nd week	3rd week	4th week	8th week
FSBM	2.0 (1.0~2.0)	2.0 (1.0~2.0)	2.00 (2.0~2.0)	3.0 (2.5~3.0)^*∗*^	4.0 (3.0~4.0)^*∗*^	3.0 (3.0~4.0)^*∗*^
BSS	2.0 (1.0~2.0)	- -	- -	- -	3.0 (3.0~4.0)^*∗*^	3.0 (3.0~3.0)^*∗*^
PAC-SYM	26.0 (24.5~32.0)	26.0 (24.0~29.0)	26.0 (24.0~31.0)	22.0 (20.0~24.0)^*∗*^	16.0 (14.0~18.5)^*∗*^	18.0 (14.5~18.5)^*∗*^
PAC-QOL	52.0 (48.0~58.0)	52.0 (48.0~57.0)	52.0 (48.0~55.0)	39.0 (35.0~43.0)^*∗*^	31.0 (27.0~36.0)^*∗*^	33.0 (26.5~38.0)^*∗*^
SAS	44.0 (37.5~51.5)	- -	- -	- -	30.0 (28.0~34.5)^*∗*^	28.0 (26.0~28.5)^*∗*^
SDS	44.0 (37.5~49.5)	- -	- -	- -	30.0 (28.0~32.5)^*∗*^	29.0 (27.0~30.5)^*∗*^

*n* = 17; ^*∗*^*P* < 0.001 versus baseline.

**Table 2:** The comparison of plasma bile acids among FC patients and control group. Median (quartile range), nonparametric test.

Bile acids (ng/ml)	Before TN treatment (treatment group, *n* = 17)	After TN treatment (treatment group, *n* = 17)	Control group (*n* = 20)
TUDCA	0.00 (0.00~1.65)	0.00 (0.00~6.22)	1.02 (0.18~2.10)
TCDCA	22.80 (7.33~55.60)	36.80 (14.90~58.95)	13.65 (7.38~47.45)
UDCA	11.80 (5.23~36.30)	26.00 (12.45~44.75)	8.74 (2.68~33.78)
CDCA	44.70 (19.15~44.70)	98.00 (19.95~273.00)	87.80 (21.60~283.35)
LCA	ND	ND	<5
GCA	43.50 (17.85~147.35)	52.60 (23.30~106.00)	16.95 (10.83~70.80)
GCDCA	118.70 (56.05~491.10)	188.20 (78.60~255.20)	400.00 (90.25~677.50)
GDCA	34.80 (9.41~89.05)	20.60 (12.40~38.50)	28.95 (11.33~52.43)
TDCA	31.30 (11.85~60.05)^#^	12.64 (9.29~19.95)^#*∗*^	2.40 (1.85~4.63)
TCA	67.60 (30.71~178.90)^#^	31.70 (12.54~72.90)^#*∗*^	2.45 (1.03~26.57)
TLCA	4.10 (3.23~4.95)^#^	3.20 (2.09~3.70)^#*∗*^	1.05 (0.26~2.26)
CA	18.70 (7.35~31.00)	21.10 (13.60~33.00)	28.70 (3.83~92.35)

^#^ *P* < 0.05 versus control group (Mann-Whitney *U* Test); ^*∗*^*P* < 0.05 versus treatment group before TN treatment (Wilcoxon Signed Ranks Test); ND, not detectable.

**Table:** (a) The Spearman correlation coefficient of the plasma gastrointestinal hormones and bile acids in FC patients before TN treatment (r-s, *P*)

	5-HT		Motilin		VIP		Somatostatin	
TDCA	−0.38	0.14	−0.05	0.86	0.09	0.72	−0.02	0.94
TCA	−0.26	0.32	0.01	0.98	0.21	0.42	−0.26	0.32
TLCA	−0.12	0.65	0.04	0.88	0.25	0.33	−0.13	0.63

*n* = 17; r-s, Spearman correlation coefficient.

**Table:** (b) The Spearman correlation coefficient of the plasma gastrointestinal hormones and bile acids in FC patients after TN treatment (r-s, *P*)

	5-HT		Motilin		VIP		Somatostatin	
TDCA	0.03	0.90	−0.19	0.46	−0.06	0.81	−0.05	0.85
TCA	−0.41	0.10	−0.29	0.25	0.20	0.44	0.41	0.10
TLCA	−0.23	0.37	−0.12	0.66	0.06	0.82	−0.19	0.46

*n* = 17; r-s, Spearman correlation coefficient.

**Table:** (c) The Spearman correlation coefficient of the plasma gastrointestinal hormones and bile acids in healthy volunteers (r-s, *P*)

	5-HT		Motilin		VIP		Somatostatin	
TDCA	−0.07	0.76	0.11	0.65	0.23	0.32	0.19	0.43
TCA	−0.33	0.15	−0.02	0.93	0.01	0.96	0.09	0.71
TLCA	−0.06	0.81	−0.10	0.69	0.06	0.80	−0.17	0.48

*n* = 20; r-s, Spearman correlation coefficient.

**Table 4:** The Spearman correlation coefficient of the level of plasma gastrointestinal hormones, the levels of plasma bile acids, and the scores of questionnaires of the FC patients before TN treatment (r-s, *P*).

	FSBM		BSS		PAC-SYM		PAC-QOL	
5-HT	−0.20	0.45	0.02	0.93	0.23	0.38	−0.12	0.65
Motilin	−0.20	0.45	0.02	0.94	0.00	0.99	−0.15	0.56
VIP	−0.01	0.96	0.40	0.11	0.06	0.83	0.10	0.71
Somatostatin	−0.07	0.78	0.21	0.41	−0.12	0.64	0.33	0.19
TDCA	0.15	0.58	−0.21	0.43	−0.33	0.19	−0.10	0.71
TCA	0.10	0.71	0.11	0.67	−0.31	0.22	−0.19	0.47
TLCA	−0.01	0.96	−0.30	0.24	0.00	1.00	−0.41	0.11

*n* = 17; r-s, Spearman correlation coefficient.
